# Transcriptomic Analysis of the Hepatopancreas in the Sex-Related Size Differences of *Macrobrachium nipponense*

**DOI:** 10.3390/vetsci11090445

**Published:** 2024-09-21

**Authors:** Yabing Wang, Guangde Qiao, Yanfeng Yue, Shiming Peng, Hongtuo Fu

**Affiliations:** 1 Key Laboratory of Marine and Estuarine Fisheries, Ministry of Agriculture, East China Sea Fisheries Research Institute, Chinese Academy of Fishery Sciences, Shanghai 200090, China; wangyabing@ecsf.ac.cn (Y.W.); qiaogd@ecsf.ac.cn (G.Q.);; 2 Key Laboratory of Freshwater Fisheries and Germplasm Resources Utilization, Ministry of Agriculture, Freshwater Fisheries Research Center, Chinese Academy of Fishery Sciences, Wuxi 214081, China

**Keywords:** *Macrobrachium nipponense*, hepatopancreas, RNA-sequencing, sex-related size differences, differentially expressed genes

## Abstract

**Simple Summary:**

The oriental river prawn, *Macrobrachium nipponense*, is a widely distributed species in freshwater and low-salinity estuarine regions of China and other Asian countries. This species has become an important commercial commodity in China because of its high nutritional value and palatability. There are significant differences in growth between male and female *M. nipponense*. The aim of this study was to determine the hepatopancreas transcriptome differences between sex-related size differences in *M. nipponense*. We identified four genes associated with sex-related size differences, as well as six closely related metabolic pathways. The results indicated the molecular mechanism underlying the sex-related size differences and identified key genes and metabolic pathways. This data will be invaluable to support explanations of individual differences between male and female prawns.

**Abstract:**

*Macrobrachium nipponense*, a commercially popular crustacean species within the Chinese context, is recognized for its exceptional nutritional composition and palatability. There are significant differences in growth between male and female *M. nipponense*. Herein, transcriptomics was used to determine the hepatopancreas transcriptome differences between sex-related size differences in *M. nipponense*. We identified 974 differentially expressed genes (DEGs) between the SHE (female) and BHE (male) groups, which were validated using RT-qPCR. The genes encoding matrix metalloproteinase-9 (*MM9*), Ribosome-binding protein 1 (*RBP1*), Aly/REF export factor 2, and hematological and neurological expressed 1 (*HN1*) may play a role in modulating the sex-related size differences observed in *M. nipponense*. Clusters of orthologous groups and gene ontology functional analysis demonstrated that the DEGs for sex-related size in *M.nipponense* were associated with various biological functions. The Kyoto Encyclopedia of Genes and Genomes pathways analysis demonstrated that upregulated DEGs were mainly enriched in lysine biosynthesis, tryptophan metabolism, and lysine degradation pathways, whereas the downregulated DEGs were mainly enriched in ascorbate and aldarate metabolism, retinol metabolism, and drug metabolism-cytochrome P450 pathways. The results indicated the molecular mechanism underlying the sex-related size differences and identified key genes. This data will be invaluable to support explanations of individual differences between male and female prawns.

## 1. Introduction

The oriental river prawn, *Macrobrachium nipponense* (Crustacea; Decapoda; Palaemonidae), is a widely distributed species in freshwater and low-salinity estuarine regions of China and other Asian countries [1,2,3]. This species has become an important commercial commodity in China because of its high nutritional value and palatability. The annual production of *M. nipponense* has exhibited a gradual increase in recent years, reaching 226,312 tons in 2022 [4]. The annual output value was approximately 2.8 billion US dollars in 2021 [3]. As is the case with other *Macrobrachium* species, there are notable differences between male and female *M. nipponense*. Males typically exhibit faster growth rates than their female counterparts, reaching larger sizes at the time of harvest each year [3]. Therefore, determining the molecular regulatory mechanisms underlying the growth and development of *M. nipponense* will enhance our understanding of sex-related size differences in prawns.

The significant difference in growth between males and females, succinctly termed male and female growth dimorphism, or more academically referenced as sexual size dimorphism (SSD), is a consistent and widely observed characteristic in prawns [5,6,7]. Predominant males possess an enhanced capacity to claim breeding territories and scavenge for sustenance, thereby perpetuating their own developmental trajectory [7]. Growth is a multifaceted quantitative characteristic, regulated by an entwined network of multiple genes. The discernment and analysis of growth-associated regulatory genes can furnish primordial data, fostering advancements in the molecular breeding of aquatic organisms. Fortuitously, a rising number of scholars are dedicating their efforts to elucidate the molecular regulatory mechanisms that underpin the growth of phenotypes of these aquatic entities. An assortment of pivotal functional genes, alongside their corresponding pathways, have been acknowledged, posing an instrumental influence in the orchestration of growth attributes [7,8,9,10]. The latest scientific inquiries have unveiled that central modulators of growth and evolution in aquatic organisms include growth hormone (GH), growth hormone receptor (GHR), and insulin-like growth factor (IGF) [11,12]. Similar outcomes were observed in *Pangasianodon hypophthalmus* [13], *Odontobutis potamophila* [14], *Oreochromis niloticus* [15], *Salmo salar* [16], *Dicentrarchus labrax* [17], *Cyprinus carpio* [18], *Pelteobagrus fulvidraco* [19], and *Larimichthys crocea* [20]. The growth of aquatic organisms is regulated by hormones secreted from the neuro-endocrine system, in addition to which other genes exert a regulatory influence. In *Ctenopharyngodon idella*, glyceraldehyde-3-phosphate dehydrogenase and myoglobin 1 showed elevated expression in the accelerated growth group [21]. These findings suggest that these enzymes may play a role in promoting muscle growth. In *Acipenser dabryanus*, it was established that the accelerated growth was markedly linked to glycolysis, protein synthesis, and antioxidant functions [22]. The expression of specific genes, including those encoding glycogen phosphorylase, heat shock protein 90, crustacean hyperglycemic hormone, cathepsin L, and peroxidasin, was observed in *Macrobrachium rosenbergii*, which were closely related to its growth [23,24,25].

Techniques such as RNA sequencing (RNA-seq) have been used extensively to identify genes linked to the growth and maturation of aquatic organisms, thereby elucidating those hereditary elements associated with growth efficiency and the proliferation of muscle fibers [7,26]. Leveraging this approach, an extensive array of functionally potent genes and enhanced pathways have been efficaciously discerned, while DEG analyses have proficiently illuminated the molecular regulatory machineries pertaining to growth in aquatic organisms [7,27]. The hepatopancreas is the most important metabolic organ of *M. nipponense* and is involved in a variety of its life processes, such as molting [28], growth [29], gonadal development [30], and the response to hypoxia [31]. Nevertheless, the growth mechanisms intrinsic to *M. nipponense* have not been intensively investigated. Consequently, the identification of functional genes and enriched pathways associated with sex-related size dynamics would provide invaluable insights for subsequent genomic studies investigating the adjustment mechanism underlying sex-related size-difference disparities.

Therefore, within the scope of this investigation, we employed next-generation sequencing in conjunction with bioinformatic methodologies to distinguish transcriptomic differences associated with sex-related size in *M. nipponense*. The objective was to identify functional genes and enriched pathways relevant to sex-related size, thereby establishing a robust theoretical framework for explaining individual differences between male and female prawns.

## 2. Materials and Methods

### 2.1. Sample Collection

Specimens of *M. nipponense*, all with a homogeneous genetic background, were obtained from the Chinese Academy of Fisheries Science, located in Wuxi City, China. The specimens of *M. nipponense* selected for study were all full-sibling offspring, descended from a single male and female progenitor, aged-matched, and nurtured within a uniform set of environmental parameters. We distinguished between males and females by examining their gonads [32]. Randomly selected males were referred to as the BHE group (5.2 ± 0.5 g) and females were referred to as the SHE group (3.1 ± 0.3 g). Thereafter, hepatopancreatic tissues (n = 3 per group) were excised rapidly for gene expression analyses, followed by their instantaneous immersion in liquid nitrogen and storage at a −80 °C until RNA extraction.

### 2.2. Total RNA Extraction and Illumina Sequencing

The extraction of total RNA from gonadal specimens was successfully accomplished utilizing the Trizol reagent (TaKaRa, Shiga, Japan), adhering strictly to previously reported procedures [24]. Instruments such as a Nanodrop 2000 spectrophotometer (Nanodrop Technologies, Wilmington, DE, USA), an Agilent 2100 bioanalyzer (Agilent Technologies, Santa Clara, CA, USA) were used to determine the purity, concentration, and integrity of the RNA samples. The subsequent construction of the cDNA libraries was conducted in accordance with the manufacturer’s guidelines, utilizing a AMPure XP Bead-Based Reagent (Beckman, Inc., Shanghai, China). The transcriptome sequencing (RNA-seq) of the libraries to produce paired-end reads was executed on the Novaseq-PE 150 platform This platform was developed by Genepioneer Biotechnologies Technology Co., Ltd. (Nanjing, China).

### 2.3. Transcriptome Sequence Assembly and Annotation

The raw reads were subjected to a series of filtration steps, including number of unknown bases N < 5, removal of sequences with base mass values less than 5 for 50% of the length of the reads and removal of splice sequences.

The clean data was aligned sequentially with the specified reference genome to generate mapped data (https://ftp.cngb.org/pub/CNSA/data2/CNP0001186/CNS0254395/CNA0014632/, accessed on 15 April 2024). All Mapped Data were searched against the NCBI non-redundant protein sequences (NR, http://ncbi.nlm.nih.gov/, accessed on 16 April 2024), Cluster of Orthologous Groups of proteins (COG, https://www.ncbi.nlm.nih.gov/COG/, accessed on 16 April 2024), Gene Ontology (GO, https://geneontology.org/, accessed on 16 April 2024), Kyoto Encyclopedia of Genes and Genome (KEGG, http://www.kegg.jp, accessed on 16 April 2024), and protein family (Pfam, http://pfam.xfam.org/, accessed on 16 April 2024) databases.

### 2.4. DEGs Analysis

In this study, DEGs were filtered through the DESeq2 (Version 1.26.0), subject to a sorting threshold of |log2 (fold change)| > 1 and Q-value < 0.05 (http://www.bioconductor.org/packages/release/bioc/html/DESeq2.html, accessed on 17 April 2024). Ultimately, the COG, GO, and KEGG enrichment analyses of DEGs were executed utilizing the Cluster Profiler software (Version 3.4.4). COG functions, GO terms, and KEGG pathways—exhibiting a False Discovery Rate (FDR) less than 0.05—were deemed as being significantly enriched.

### 2.5. Quantitative Real-Time Reverse Transcription PCR Validation (RT-qPCR)

To validate the RNA-seq data, 17 DEGs were selected for RT-qPCR analysis. PCR primers (Table S1) were designed using a Primer designing tool (https://www.ncbi.nlm.nih.gov/tools/primer-blast/, accessed on 17 April 2024).

RNA was meticulously extracted and subsequently reverse transcribed into cDNA. The qPCR reactions were performed using the SuperRT cDNA Synthesis Kit (Cwbio, Taizhou, China), with the *EIF* gene (encoding eukaryotic initiation factor) as the internal control [24]. In order to reduce the likelihood of experimental inaccuracy, each specimen was examined in triplicate. These investigative measures were conducted using a CFX96 Touch™ qRT-PCR instrument (Bio-Rad Laboratories, Hercules, CA, USA). The relative gene expression was assessed utilizing the 2^−ΔΔCT^ technique.

## 3. Results

### 3.1. Comparative Analysis of Sexual Size Dimorphism of M. nipponense

**Figure 1:**
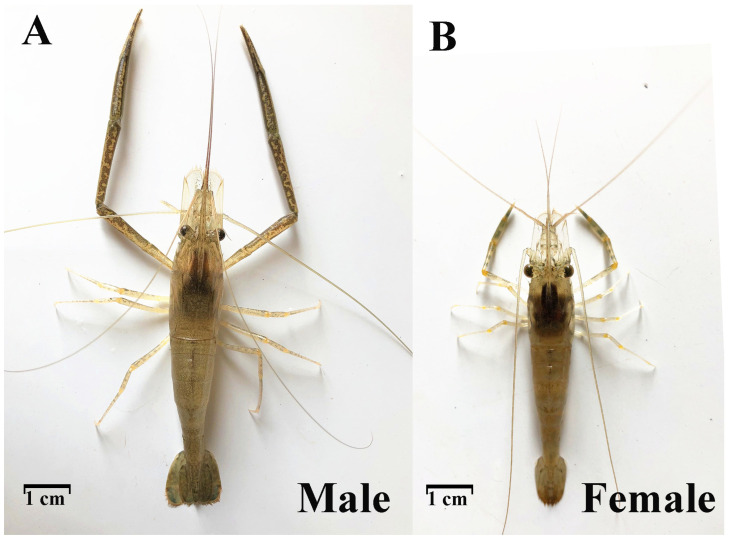
Sexual size dimorphism in *M. nipponense*.

Under the same aquaculture environment, males and females of the same batch of *M. nipponense* differed significantly from each other, showing obvious SSD (Figure 1).

### 3.2. Transcriptome Profiles and Annotation

The *M. nipponense* RNA library sequencing generated 146,771,796 raw reads in total (Table 1). The clean data of each sample was ≥5.95 Gb, and the percentage of Q30 bases was ≥89.85%.

The abbreviated sequences derived from the RNA-sequencing dataset were accurately aligned to the *M. nipponense* genome using HISAT2 (http://daehwankimlab.github.io/hisat2, accessed on 17 April 2024) and the percentage of uniquely mapped transcripts increased from 88.41 to 90.84%. Finally, we constructed a stringent set of *M. nipponense* RNA transcripts comprising 47,712 annotated protein-coding genes.

### 3.3. Correlation Analysis between Samples

**Figure 2:**
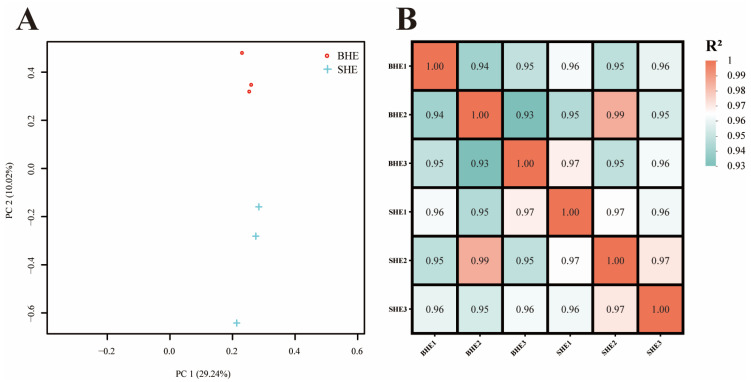
Relationship analysis of sexual size dimorphism in *M. nipponense*. (**A**) Principal component analysis (PCA) of different transcriptome samples; (**B**) Inter-sample correlation coefficient heatmap.

The outcomes derived from the Principal Component Analysis (PCA) executed on the transcriptome samples demonstrated that sex-related size discrepancies were the paramount element influencing transcriptomic data. Subsequently, the sequenced specimens were divided into two distinct subcategories based on their sex-related size (Figure 2A). The heatmap show that the clustering results were similar to those of the PCA analysis (Figure 2B).

### 3.4. DEGs Function Analysis

**Figure 3:**
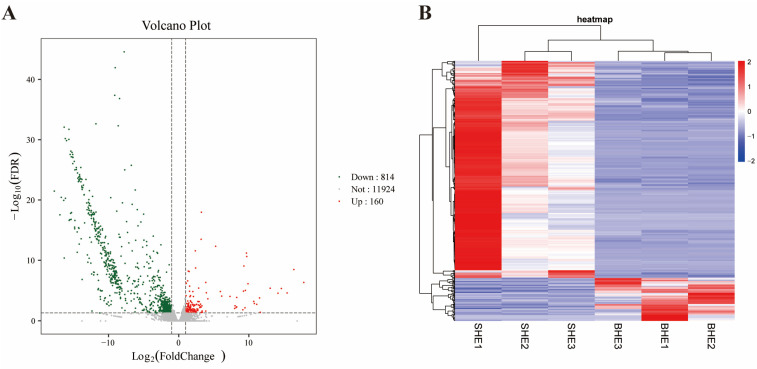
(**A**) Volcano plot of DEGs between SHE and BHE groups. (**B**) Heatmap of DEGs between the SHE and BHE groups. DEG, differentially expressed gene; FDR, false discovery rate.

A volcano plot was used to show the DEGs between SHE and BHE groups, identified according to the fold change value (Figure 3A). A total of 974 DEGs were screened between SHE and BHE groups; 814 DEGs were downregulated and 160 DEGs were upregulated. Hierarchical clustering analysis was performed on the screened DEGs to cluster genes with the same or similar expression behavior, as shown in Figure 3B. The top 20 genes in the upregulated and downregulated groups were selected for analysis, and it was determined that only 17 genes had clear functional annotations (Table 2).

### 3.5. Clusters of Orthologous Groups (COG) Functional Annotations of the DEGs

**Figure 4:**
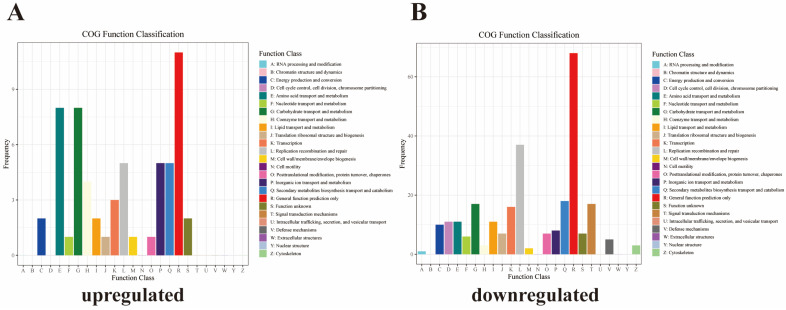
Clusters of orthologous groups (COG) functional annotations of DEGs (**A**) upregulated DEGs; (**B**) downregulated DEGs.

A total of 59 upregulated DEGs were annotated to 15 metabolic pathways in the COG functional annotation analysis. Most of the DEGs were annotated in general function prediction only (11 DEGs), followed by carbohydrate transport and metabolism (5 DEGs), and amino acid transport and metabolism (5 DEGs) (Figure 4A). In addition, 269 downregulated DEGs were annotated to 20 pathways by COG functional annotation. Most of the DEGs were annotated in general function prediction only (68 DEGs), followed by replication, recombination and repair (37 DEGs) and secondary metabolites biosynthesis, transport, and catabolism (18 DEGs) (Figure 4B).

### 3.6. Gene Ontology (GO) Analyses of DEGs

**Figure 5:**
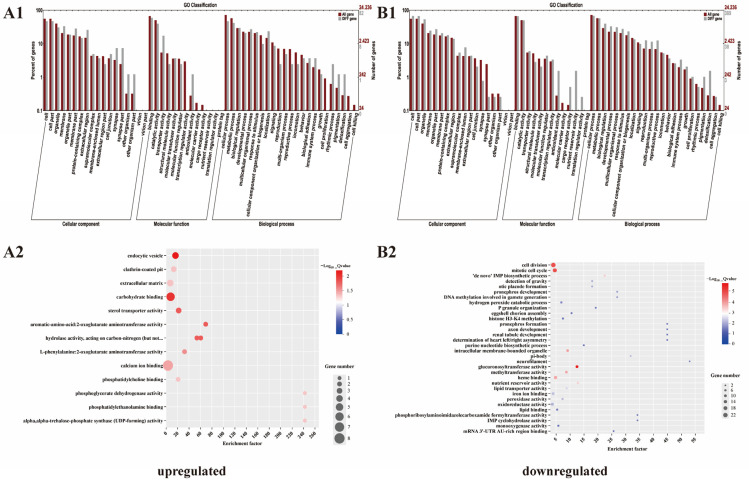
Enriched gene ontology (GO) terms associated with the DEGs. (**A1**,**A2**) upregulated DEGs; (**B1**,**B2**) downregulated DEGs.

GO enrichment (Figure 5) categorized all DEGs into three groups: molecular functions, cellular components, and biological processes. A total of 43 GO terms (for the upregulated DEGs) and 49 GO terms (for the downregulated DEGs) were enriched, among which the highest classification terms were “binding” (48 DEGs) and “cellular process” (262 DEGs), respectively (Figure 5(A1,B1)). Further examination revealed that the upregulated DEGs were enriched in “endocytic vesicles”, “carbohydrate binding”, and “sterol transporter activity”. Conversely, the downregulated DEGs were enriched in “glucuronosyltransferase activity”, “cell division”, and “mitotic cell cycle” (Figure 5(A2,B2)).

### 3.7. KEGG Enrichment Analyses of DEGs

**Figure 6:**
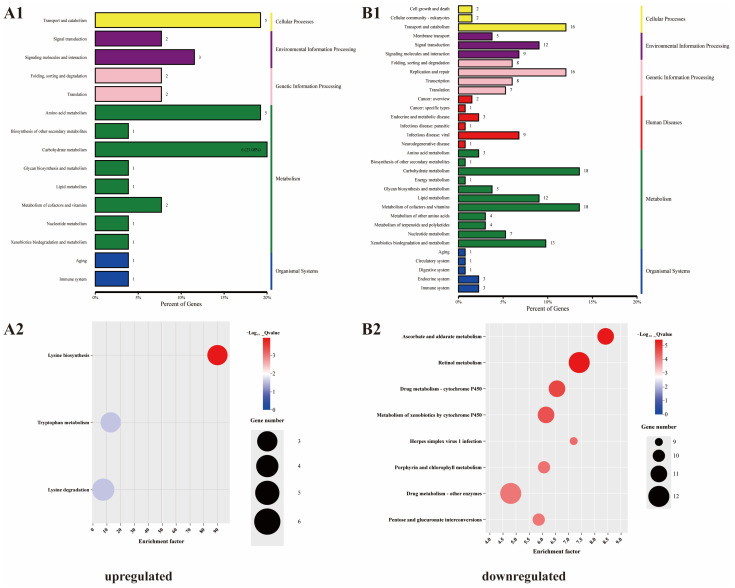
Enriched Kyoto Encyclopedia of Genes and Genomes (KEGG) pathways associated with the DEGs. (**A1**,**A2**) upregulated; (**B1**,**B2**) downregulated.

**Figure 7:**
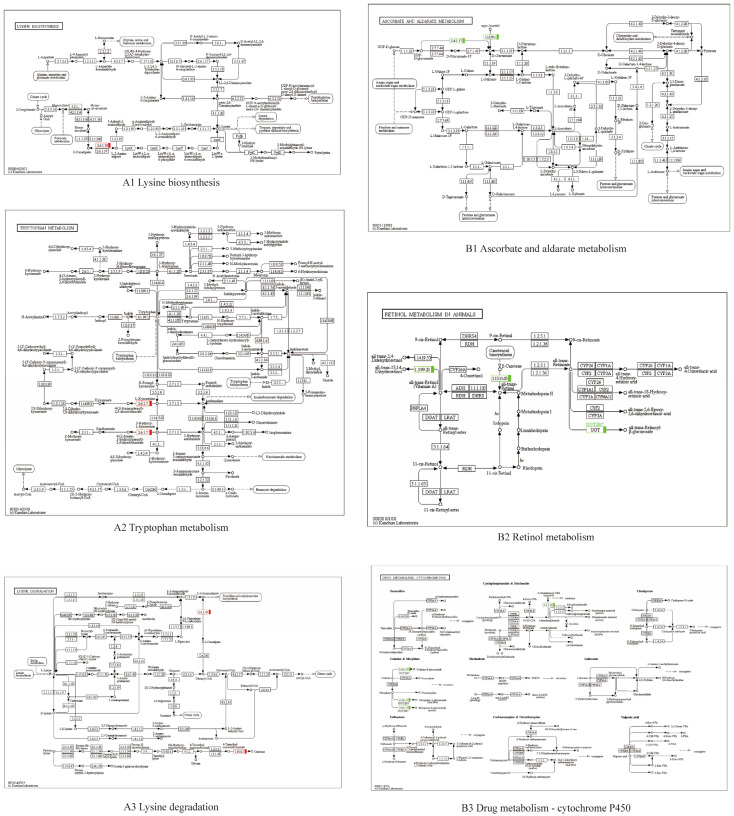
KEGG enrichment of different metabolism pathway between SHE and BHE groups. (**A1**–**A3**) upregulated; (**B1**–**B3**) downregulated.

KEGG analysis was used to determine the enrichment of the DEGs for metabolic pathways. The upregulated DEGs were enriched for 16 pathways and the downregulated DEGs were enriched for 33 pathways. In total, six types of KEGG pathway were identified in the downregulated group. In addition to pathways related to human diseases, other types of pathways were also associated with the upregulated group; however, there were differences in the degree of enrichment (Figure 6(A1,B1)). Further examination revealed that the upregulated DEGs were significantly enriched in “lysine biosynthesis”, “tryptophan metabolism”, and “lysine degradation”. Conversely, the downregulated DEGs were significantly enriched in “ascorbate and aldarate metabolism”, “retinol metabolism”, and “drug metabolism-cytochrome P450” (Figure 6(A2,B2) and Figure 7).

### 3.8. RT-qPCR Verification of Transcriptomic Data

**Figure 8:**
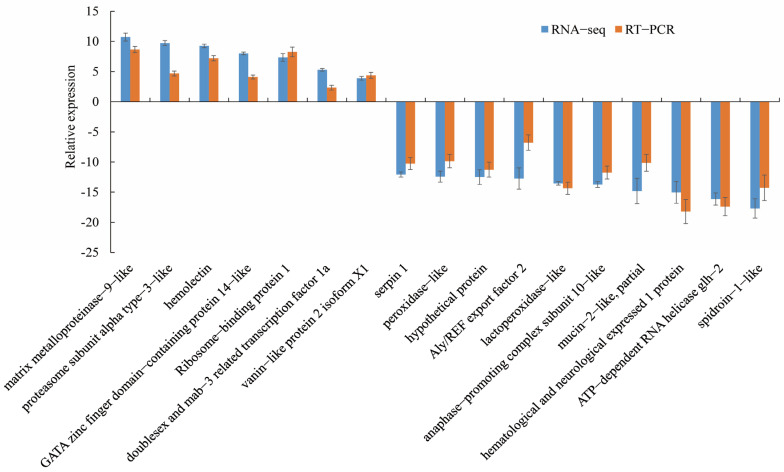
Verification of DEGs in *M. nipponense.* RNA-seq, RNA sequencing; RT-qPCR, quantitative real-time reverse transcription PCR.

The 17 DEGs with the most significant differences were selected, among which 7 were upregulated and 10 were downregulated. Concordance between the RT-qPCR and RNA-seq data-based mRNA expression was assessed for the 17 selected genes, and the results of the two analyses were consistent for 15 genes (Figure 8).

## 4. Discussion

Body weight is an important factor in the selection of animals for genetic improvement [7,33]. Nevertheless, the codified mechanism orchestrating body weight regulation continues to elude comprehension, an ambiguity which subsequently restrains the potential for genetic enhancement of these aquatic animals, thereby eliciting a ripple effect on the progression of aquacultural yield [7,34]. Male *M. nipponense* grow faster and are larger at harvest than females [35]. Therefore, we identified the DEGs between the different sexes to identify size-related difference genes. The comparison of transcription levels between sex-specific size differences enables the identification of DEGs that can be exploited to gain mechanistic insight into the underlying biological processes involved. The sexually dimorphic size-related DEGs identified in this study may be useful in revealing individual differences between male and female prawns.

In this paper, DEGs such as *MMP-9*, *RBP1*, Aly/REF export factor 2, and *HN1* were identified through hepatopancreas transcriptomic analysis. MMP-9 represents a category defined as matrixins, a distinctive class of enzymes related to the zinc-metalloproteinases family. These are instrumental in orchestrating the degradation process of the extracellular matrix [36]. MMP family-mediated extracellular matrix breakdown is associated with normal physiological processes, such as embryonic development, reproduction, angiogenesis, bone development, and cell migration [37,38]. Studies have shown that ribosome-binding proteins are essential for embryonic development, and regulation of the cell cycle and proliferation [39]. The Aly/REF export factor, a universally expressed nuclear protein, operates as a molecular guardian and export mediator, playing a pivotal role in the nuclear export of both spliced and unspliced mRNA. Integral to this process is the Transcription-Export (TREX) complex, a master regulator in mRNA export that comprises the THO subcomplex, the RNA helicase UAP56, and the RNA-binding protein Aly [40,41,42]. Hematological and neurological expressed 1 protein, a member of the Notch family, is encoded by the *HN1* gene [43]. Members of the Notch family serve crucial roles in numerous developmental sequences by governing decisions pertaining to cellular destiny [44]. The Notch signaling circuit represents an evolutionarily preserved intercellular transmission pathway, which oversees interactions between physically neighboring cells [45]. Particularly in Drosophila, the establishment of an intercellular communicative pathway through Notch interaction with its cellular ligands, Delta and Serrate, orchestrates a pivotal role in the organism’s developmental process [46,47]. In the present study, the genes that code for *MMP 9*, *RBP1*, Aly/REF export factor 2, and *HN1* displayed significant differential expression between the SHE and BHE assemblies. This intimates that these DEGs could potentially contribute to modulating the sex-related size differences of *M. nipponense*.

Herein, the results of COG and GO analyses helped to reveal the mechanisms underlying *M. nipponense* growth. The upregulated DEGs were enriched in carbohydrate and amino acid metabolism. Carbohydrate metabolism represents the biochemical processes responsible for the metabolic formation, breakdown, and interconversion of carbohydrates in living organisms [48,49]. Carbohydrates are essential to many essential metabolic pathways [48]. When animals and fungi consume plants, they use cellular respiration to break down the stored carbohydrates to provide energy to cells [48]. Both animals and plants temporarily store the released energy in the form of high-energy molecules, such as ATP, for use in various cellular processes [50]. Amino acid metabolism refers to the biochemical processes that produce, break down, and use amino acids [51]. The body employs amino acids to synthesize a multitude of vital molecules, including proteins, enzymes, hormones, and other essential compounds. Additionally, amino acids serve as a precursor for glucose, a crucial source of energy for the body [51]. The body can also use amino acids to make lipids (fats) and cholesterol [52]. An organism can promote growth by regulating carbohydrate and amino acid metabolism [53,54,55,56].

The KEGG enrichment analysis showed that the upregulated DEGs were enriched in lysine biosynthesis and degradation, and tryptophan metabolism pathways. Lysine plays several roles, most importantly in protein synthesis, but also in the cross-linking of collagen polypeptides, in the absorption of essential minerals, and in the production of carnitine, which is vital for fatty acid metabolism [57,58]. From a regulatory perspective, lysine is located at the top level of control, affecting other amino acid metabolisms. Lysine can contribute to the metabolism of other nutrients, such as Ca and cholesterol [57]. The impact of dietary lysine on hormone production and activity is reflected in the alteration of plasma concentrations of insulin and insulin-like growth factor 1. Lysine residues in peptide chains represent crucial sites for post-translational modification (PTM), which is involved in histone modification and epigenetic regulation of gene expression. Beyond its involvement in PTM, lysine concurrently plays a role in the post-transcriptional phase of protein manifestation [58]. Tryptophan is an α-amino acid that is used in the biosynthesis of proteins [59]. Tryptophan is also a precursor of the neurotransmitter serotonin, the hormone melatonin, and vitamin B3 [60]. Dietary tryptophan might affect animal growth, such as in *Mus musculus* [61], *Sus scrofa domestica* [62], *Gallus gallus* [63], and *Salmo gairdneri* [64]. In *Eriocheir sinensis*, L-tryptophan promoted cheliped regeneration through melatonin, serotonin. and dopamine involvement [65].

The downregulated DEGs were enriched in “ascorbate and aldarate metabolism”, “retinol metabolism”, and “drug metabolism-cytochrome P450” pathways. Ascorbate and aldarate metabolism is a crucial carbohydrate metabolic pathway that can prevent cells from oxidative damage [66]. Ascorbic acid is an essential antioxidant and plays an essential role in growth [67,68]. Under crowding stress, the ascorbate and aldarate pathway was determined to affect the growth of hybrid sturgeon [68]. Additionally, ascorbate and aldarate metabolism was enriched in shrimp fed diets with different Se content, resulting in significant effects on shrimp growth [69]. Retinol metabolism is critical for many physiological processes, including embryonic development, reproduction, differentiation, and maintenance of various epithelia [70,71]. In rats, cytochrome P450 regulates growth hormone secretion and exhibits sexual dimorphism [72]. In contrast with the findings of this study, ascorbate and aldarate metabolism, drug metabolism-cytochrome P450, and retinol metabolism pathways were inhibited in Angus beef longissimus muscle during the growth phase [73]. These pathways can reveal the most important DEGs and provide clues to further understand the sex-related size differences in *M. nipponense* growth.

In recent years, there has been a growing recognition of the unstable production of *M. nipponense*. The emergence of early puberty and growth retardation as significant constraints on the development of *M. nipponense* aquaculture has also become increasingly evident [74]. This study used transcriptomic profiling technology to investigate the expressed hepatopancreas-related genes and identified gene candidates associated with sexually dimorphic size based on the existing theories and research results. The results of this research may also provide a theoretical basis for explaining individual differences between male and female prawns.

## 5. Conclusions

In the presented study, a transcriptome profiling analysis was performed to investigate the sex-related size differences in *M. nipponense*. The most important genes and pathways significantly associated with sex-related size were identified, and their functions were primarily related to tryptophan metabolism pathways and lysine biosynthesis and degradation pathways. The present study provides valuable fundamental information for further research on the molecular mechanism of individual differences between male and female prawns.

## Figures and Tables

**Table 1:** Data quality for each sample.

Sample	Raw Reads	Clean Reads	GC (%)	Q20 (%)	Q30 (%)	Reads Aligned
BHE1	25,602,059	7,680,617,700	44.2	96.65	90.94	45764142 (89.38%)
BHE2	19,817,043	5,945,112,900	44.28	96.62	90.85	35522900 (89.63%)
BHE3	22,983,339	6,895,001,700	44.23	96.95	91.47	41631683 (90.57%)
SHE1	26,175,015	7,852,504,500	44.08	96.79	91.25	47555729 (90.84%)
SHE2	27,515,937	8,254,781,100	43.68	96.42	90.46	49625449 (90.18%)
SHE3	24,678,403	7,403,520,900	44.32	96.51	90.68	43633973 (88.41%)

**Table 2:** Expression of key DEGs between SHE and BHE groups.

Gene Id	log_2_ Fold Change
matrix metalloproteinase-9-like	10.72295443
proteasome subunit alpha type-3-like	9.72755699
hemolectin	9.257651261
GATA zinc finger domain-containing protein 14-like	8.010151712
Ribosome-binding protein 1	7.341541976
doublesex and mab-3 related transcription factor 1a	5.293209029
vanin-like protein 2 isoform X1	3.889522683
serpin 1	−12.07129799
peroxidase-like	−12.41822183
hypothetical protein	−12.4872916
Aly/REF export factor 2	−12.73306459
lactoperoxidase-like	−13.5386898
anaphase-promoting complex subunit 10-like	−13.72725897
mucin-2-like, partial	−14.79593353
hematological and neurological expressed 1 protein	−15.03408516
ATP-dependent RNA helicase glh-2	−16.12929411
spidroin-1-like	−17.6950176

## Data Availability

The RNA-seq data have been deposited in the NCBI Short Read Archive (SRA) under accession number PRJNA1056262 (https://www.ncbi.nlm.nih.gov/bioproject/PRJNA1056262, accessed on 17 April 2024).

## Supplementary Materials

The following supporting information can be downloaded at: https://www.mdpi.com/article/10.3390/vetsci11090445/s1, Table S1: Sequences of primers used for RT-qPCR. Table S2: All the differentially expressed genes.
