# Supplementary material for: Transcriptomic Analysis of the Hepatopancreas in the Sex-Related Size Differences of Macrobrachium nipponense
Source: Vet Sci. 2024 Sep 21;11(9):445. doi: 10.3390/vetsci11090445 (PMC11435631; doi:10.3390/vetsci11090445)
Supplement: Supplementary file 1 [file vetsci-11-00445-s001.zip › Table S1 Sequences of primers used for qRT-PCR..pdf]

Table S1 Sequences of primers used for qRT-PCR.

Primer name		Sequence(5′→3′)
matrix metalloproteinase-9-like	F	GGACCTCCACTGTTTCTTT
	R	CAAAGAGGAAGGAGGAAGG
proteasome subunit alpha type-3-like	F	CAAGCAGAATGCCAAGACAGAAA
	R	GACAGTTCCAACTCAAAGTGACG
hemolectin	F	GTCATCCACTCCCGACAGGC
	R	AAGGCGAATCGAACGCGG
GATA zinc finger domain-containing protein 14-like	F	ACGGACGGACGACGGA
	R	GTGTCCTGGAGTTGGAGTCG
Ribosome-binding protein 1	F	CACACTCACTCTGCCCCATT
	R	ACGCAAAGGAATGGTAGGGG
doublesex and mab-3 related transcription factor 1a	F	CAAGACTGTCCCTGCGAACT
	R	TTCGTTGTGCTGTTTGCTCG
vanin-like protein 2 isoform X1	F	CTCTTTCAAGGACCCCCTCG
	R	AGCGACGTCCGAAATGAGTT
serpin 1	F	CAGATACGGCGAATCGGTGA
	R	CCTCCTGGAGATTAGCAGCG
peroxidase-like	F	GCTCTCAGGTCTACGGCATC
	R	GTTCTGCCTTGTTGCACTCG
hypothetical protein	F	TGGCCTGCTTTATGCTGACA
	R	ACAAGTACCGTGCCAATCGT
Aly/REF export factor 2	F	TTGGTACCGCCTATGTCGTG
	R	TTTGGTCCACCCTGAAGACG
lactoperoxidase-like	F	TCTACAACGAAGCAAGGCGT
	R	GACGGCGAAGGAGTTGAGAA
anaphase-promoting complex subunit 10-like	F	ATCCGCTGTGGGACACATTT
	R	TGTATGTGCGTACAGGACGG
mucin-2-like, partial	F	GATGCCTCGACGTCGTTTTC
	R	ACTTGGCGGTACCAAGATCG
hematological and neurological expressed 1 protein	F	CAGGTGGAGGACATACGGAC
	R	TGTTCACATTCGTGCCCTCA
ATP-dependent RNA helicase glh-2	F	ATCCTGGATTTGGCAACGGT
	R	AACTCAACATTGGCGTTGGC
spidroin-1-like	F	GCATATGCGGCTGACCTTTG
	R	AGAGTGGACATTCGAAGCGG
	F	GTTGTATGCAGTCGGCCATATTT
EIF	R	TGTCCTGAAGGTGGTGATAATGA
